# Tooth Discoloration After Regenerative Endodontic Therapy in Immature Permanent Teeth: A Systematic Review

**DOI:** 10.7759/cureus.107948

**Published:** 2026-04-29

**Authors:** Ethar Mahmoud, Safaa Beshir Omer Beshir, Niroz T Wadhaj, Mohamed Gangari, Mona Hussein, Maha Mustafa, Zeinab M Houjeiry, Doaa Mbrouk, Asim Ahmed

**Affiliations:** 1 Faculty of Dentistry, University of Khartoum, Khartoum, SDN; 2 Faculty of Dentistry, National University, Khartoum, SDN; 3 Dentistry, Al Nahda College, Khartoum, SDN; 4 Orthodontics and Dentofacial Orthopaedics, Faculty of Dentistry, University of Khartoum, Khartoum, SDN; 5 Dentistry, Beirut Arab University, Beirut, LBN; 6 Dentistry, University of Science and Technology, Riyadh, SAU; 7 Medicine and Surgery, University of Gezira, Wad Madani, SDN

**Keywords:** immature permanent teeth, regenerative endodontics, regenerative endodontic therapy, revascularization, tooth discoloration

## Abstract

Tooth discoloration is a common esthetic complication following regenerative endodontic therapy in immature permanent teeth. This systematic review aimed to summarize the reported occurrence of post-treatment discoloration, identify protocol-related contributors, and synthesize preventive strategies. A comprehensive literature search was conducted, and eligible clinical studies were included after screening and full-text assessment. Due to substantial heterogeneity across study designs, regenerative protocols, discoloration assessment methods, and follow-up schedules, quantitative pooling was not appropriate. Across the included evidence, reported discoloration varied widely, with higher occurrence described in some trauma-focused cohorts and lower occurrence reported in selected material-comparison studies. The evidence indicates that discoloration risk is influenced by modifiable clinical factors, particularly coronal barrier material selection and access cavity or pulp chamber management, and may occur even when clinical and radiographic outcomes are otherwise favorable. Overall, preventive strategies that prioritize lower-staining materials and technique-sensitive chamber decontamination appear most relevant for minimizing esthetic compromise while maintaining the biological objectives of regenerative care.

## Introduction and background

Immature permanent teeth with necrotic pulp are challenging to manage because thin dentinal walls and open apices increase fracture risk and limit the effectiveness of conventional obturation. In this clinical context, post-treatment discoloration is especially important because many affected teeth are anterior teeth in young patients, where esthetic change may influence satisfaction even when biological healing is achieved. Early clinical reports introduced pulp revascularization as a biologically oriented approach that prioritizes canal disinfection and, in some cases, supports continued root development in immature teeth [1]. Subsequent work proposed and refined protocol elements for revascularization in necrotic immature teeth, establishing the conceptual basis for contemporary regenerative approaches [2]. This evolution culminated in regenerative endodontic therapy (RET), which aims to enable ongoing root maturation and periapical healing through tissue responses after canal decontamination [3].

Contemporary guidance defines successful RET primarily by resolution of symptoms and radiographic evidence of apical healing, together with signs of continued root development, including increased root length, thickening of dentinal walls, and progressive apical closure [4]. Professional consensus statements similarly emphasize case selection, adequate disinfection, scaffold establishment, and an effective coronal seal as core determinants of predictable outcomes [5]. Despite these recommendations, substantial heterogeneity persists in published RET protocols, particularly regarding irrigation and disinfection regimens, intracanal medicaments, scaffold choice, and coronal barrier materials. This heterogeneity complicates cross-study comparison and may contribute to inconsistent reporting of both outcomes and adverse events [6].

Among adverse outcomes, tooth discoloration has emerged as a clinically important complication of RET, especially for anterior teeth, where esthetics strongly influence patient satisfaction. Reviews of unfavorable outcomes and failed cases have highlighted that esthetic complications can occur even when biological objectives are otherwise achieved [7]. Medicament-related discoloration is frequently linked to staining-prone components of antibiotic paste-based revascularization protocols, with measurable crown color changes demonstrated experimentally [8]. Clinical technique modifications aimed at minimizing coronal staining, including procedural steps that reduce coronal exposure to discoloring components, have been reported to reduce the risk or severity of discoloration while maintaining therapeutic intent [9].

Biomaterial-related discoloration is also consistently implicated, particularly with mineral trioxide aggregate formulations used as coronal barriers, where intrinsic material properties and interactions with dentin may contribute to crown darkening [10]. A broader body of evidence indicates that multiple endodontic materials can induce discoloration, reinforcing the importance of material selection and placement strategy in esthetic risk management in RET [11]. Evidence syntheses and comparative reviews of regenerative endodontic interventions also emphasize variability in clinical and radiographic outcomes and underscore the need to characterize complications and their determinants alongside measures of biological success [12]. However, discoloration remains inconsistently reported and is often treated as a secondary or adverse outcome rather than a primary esthetic endpoint, which limits clear interpretation of its incidence and protocol-related contributors. Therefore, the primary objective of this systematic review is to synthesize the reported incidence of tooth discoloration following RET in immature permanent teeth. Secondary objectives are to identify protocol-related contributors to discoloration through prioritized comparison of coronal barrier materials, intracanal medicament strategies, scaffold type, and access cavity or pulp chamber management, and to summarize prevention strategies that minimize discoloration risk while preserving the biological aims of RET.

## Review

Methods

Study Design and Reporting Standards

This systematic review was conducted and reported in accordance with the Preferred Reporting Items for Systematic Reviews and Meta-Analyses (PRISMA) 2020 statement [13]. Eligibility criteria were formulated using the PICOS framework (Population, Intervention, Comparison, Outcomes, and Study Design) (Table 1) [14].

**Table 1:** The PICOS framework was used to define eligibility criteria for the systematic review PICOS, Population, Intervention, Comparison, Outcomes, and Study Design; RET, regenerative endodontic therapy; REP, regenerative endodontic procedure; MTA, mineral trioxide aggregate; PRP, platelet-rich plasma; PRF, platelet-rich fibrin; A-PRF, advanced platelet-rich fibrin.

PICOS element	Definition in this review	
Population	Human participants with immature permanent teeth (open apices) and necrotic pulps, with or without apical periodontitis, including traumatized and non-traumatized teeth	
Intervention	Regenerative endodontic therapy (RET) or regenerative endodontic procedures (REPs), including revascularization or revitalization protocols, regardless of variations in irrigants, intracanal medicaments, scaffold type, or coronal barrier material	
Comparison	Any comparator or no comparator, including alternative RET protocols, different intracanal medicaments, different scaffold strategies (blood clot, PRP, PRF, A-PRF), different coronal barrier materials (white MTA or alternative bioceramics), or apexification when applicable	
Outcomes	Primary outcome: post-treatment tooth discoloration reported as incidence (n/N, %), severity, or measurable color change (visual assessment, shade guide, patient/parent perception, or objective instruments such as spectrophotometry). Secondary outcomes: clinical success, radiographic healing, and indicators of continued root development (root length, dentinal wall thickness, apical closure, or radiographic root area) when reported	
Study design	Original clinical studies including randomized controlled trials, non-randomized comparative studies, prospective cohorts, retrospective cohorts, and observational clinical studies with extractable outcome data. Exclusions: in vitro studies, animal studies, narrative reviews, systematic reviews, editorials, conference abstracts without full data, and single case reports	

Eligibility Criteria

We included original clinical studies evaluating RET or regenerative endodontic procedures (REPs) in immature permanent teeth, where tooth discoloration was reported as an outcome (primary, secondary, or adverse event). Because discoloration was inconsistently reported across RET studies, it was unclear whether it was recorded as a primary or secondary outcome or as an adverse event; however, this variability was recognized as a potential source of heterogeneity in outcome reporting and considered during the narrative synthesis. Eligible study designs included randomized controlled trials, non-randomized comparative studies, prospective cohorts, retrospective cohorts, and observational clinical studies with extractable outcome data. Studies were eligible if they enrolled human participants and reported follow-up sufficient to identify post-treatment color changes.

We excluded in vitro studies, animal studies, narrative reviews, systematic reviews, editorials, conference abstracts without full data, and studies where discoloration could not be extracted or was not reported. Studies focusing exclusively on mature teeth, non-regenerative apexification protocols without a regenerative component, or outcomes unrelated to tooth color were excluded.

Information Sources and Search Strategy

A comprehensive literature search was undertaken using PubMed/MEDLINE, Scopus, Web of Science, and Embase, together with supplementary manual searching. The search covered studies published from database inception to February 7, 2026, corresponding to the final pre-submission search used for the submitted draft. No language restrictions were applied, unless otherwise limited by database indexing or full-text availability. Supplementary manual searching included screening the reference lists of eligible studies and relevant review articles to identify additional clinical reports. No separate gray literature database search was performed, and conference abstracts without full data were excluded. The search strategy was designed to capture variations in RET terminology, immature tooth conditions, and discoloration outcomes (Table 2).

**Table 2:** Database search strategy and core keywords used for study identification The search syntax was adapted per database using relevant subject headings where available. Truncation (*) and phrase searching were applied where supported. RET: regenerative endodontic treatment.

Database	Search fields	Core search concept	Example search string (adapted per database)
PubMed/MEDLINE	Title/Abstract; subject headings where applicable	RET AND immature teeth AND discoloration	(("regenerative endodontic" OR "regenerative endodontic therapy" OR "regenerative endodontic procedure*" OR revascularization OR revitalization) AND (immature OR "immature permanent teeth" OR "open apex" OR "open apices" OR "necrotic pulp") AND (discolor* OR stain* OR "tooth discoloration" OR "crown discoloration" OR "color change" OR "colour change"))
Scopus	Title, Abstract, Keywords	RET AND immature teeth AND discoloration	TITLE-ABS-KEY(("regenerative endodontic" OR "regenerative endodontic therapy" OR "regenerative endodontic procedure*" OR revascularization OR revitalization) AND (immature OR "immature permanent teeth" OR "open apex" OR "open apices" OR "necrotic pulp") AND (discolor* OR stain* OR "tooth discoloration" OR "crown discoloration" OR "color change" OR "colour change"))
Web of Science	Topic	RET AND immature teeth AND discoloration	TS=(("regenerative endodontic" OR "regenerative endodontic therapy" OR "regenerative endodontic procedure*" OR revascularization OR revitalization) AND (immature OR "immature permanent teeth" OR "open apex" OR "open apices" OR "necrotic pulp") AND (discolor* OR stain* OR "tooth discoloration" OR "crown discoloration" OR "color change" OR "colour change"))
Embase	Title/Abstract; subject headings where applicable	RET AND immature teeth AND discoloration	('regenerative endodontic':ti,ab OR 'regenerative endodontic therapy':ti,ab OR revascularization:ti,ab OR revitalization:ti,ab) AND (immature:ti,ab OR 'open apex':ti,ab OR 'immature permanent teeth':ti,ab OR 'necrotic pulp':ti,ab) AND (discolor*:ti,ab OR stain*:ti,ab OR 'tooth discoloration':ti,ab OR 'crown discoloration':ti,ab OR 'color change':ti,ab OR 'colour change':ti,ab)

Study Selection Process

After duplicate removal, titles and abstracts were screened independently by two reviewers. Full-text articles considered potentially eligible were then assessed independently against the predefined eligibility criteria. Any disagreements during title and abstract screening or full-text assessment were resolved through discussion and consensus. 

Outcomes of Interest

The primary outcome was post-treatment tooth discoloration, extracted as incidence (n/N, %) at the reported follow-up timepoint(s). When available, discoloration data were recorded at each reported follow-up point; for narrative comparison, the latest available follow-up was preferentially summarized to reflect the maximum clinically relevant discoloration burden.

Secondary outcomes were extracted to support interpretation and clinical context, including clinical success (absence of pain, swelling, sinus tract, or tenderness), radiographic healing (resolution of apical radiolucency), and indicators of continued root development (changes in root length, root thickness, apical closure, or radiographic root area).

Data Extraction and Management

Data were extracted using a standardized extraction form developed in accordance with the review objectives. Before extraction, the reviewers agreed on the key data items and outcome definitions to ensure consistent recording across studies. Extraction was performed independently by two reviewers and then cross-checked for accuracy and completeness. Disagreements or unclear data items were resolved through discussion and consensus. Extracted variables included study characteristics (author, year, country, study design), participant characteristics (age range, sample size), tooth characteristics (tooth type and etiology such as trauma versus non-trauma), procedural protocol details (irrigation regimen and concentrations, intracanal medicaments, scaffold type, coronal barrier material, restorative or sealing approach, and anti-discoloration steps such as chamber cleaning or access cavity management), follow-up duration, method used to measure discoloration (subjective visual assessment, shade guides, patient/parent perception, or objective tools such as spectrophotometry), and reported discoloration outcomes.

When discoloration was not reported directly as a percentage, incidence was calculated from the reported numerator and denominator, where possible. When discoloration was reported only qualitatively or without an extractable denominator, it was retained as narrative evidence and was not converted into an incidence estimate. Because outcome definitions and assessment methods varied, raw outcomes were extracted as reported by the study authors without reclassification.

Risk of Bias Assessment

Risk of bias was assessed at the study level using tools appropriate to the study design. Before the final appraisal, the reviewers aligned their interpretations of the appraisal domains through discussions of the tool criteria and a pilot assessment of selected studies. Disagreements in judgment were resolved through discussion and consensus. Formal inter-rater agreement statistics were not calculated. Randomized controlled trials were assessed using the Cochrane Risk of Bias 2 (RoB 2) tool [15] across key domains, including the randomization process, deviations from intended interventions, missing outcome data, outcome measurement, and selective reporting. Non-randomized comparative studies and observational cohort studies were evaluated using the ROBINS-I tool, which assesses bias due to confounding, participant selection, intervention classification, deviations from intended interventions, missing data, outcome measurement, and selective reporting. Risk of bias judgments were summarized as low, some concerns, or high risk for randomized designs, and as low, moderate, serious, or critical risk for non-randomized designs, in line with tool guidance. Case series were appraised using the Joanna Briggs Institute (JBI) Critical Appraisal Checklist for Case Series [16]. Risk of bias judgments were not used to exclude eligible studies; instead, they were used to guide interpretation, with greater emphasis placed on randomized and comparative evidence when evaluating protocol-related effects and more cautious interpretation applied to uncontrolled observational and case series evidence.

Data Synthesis Approach

A meta-analysis was not performed due to substantial heterogeneity in study designs, clinical protocols, methods for discoloration measurement, and follow-up time points. Therefore, findings were synthesized using a structured narrative synthesis, with outcomes summarized quantitatively where possible using incidence (n/N, %) and described alongside protocol features. To ensure transparent reporting of narrative synthesis without meta-analysis, outcomes were synthesized according to best-practice principles emphasizing explicit grouping and structured comparison.

Studies were grouped for interpretation based on clinically meaningful protocol domains associated with discoloration risk, including scaffold type (blood clot, PRF (platelet-rich fibrin), PRP (platelet-rich plasma), A-PRF (advanced platelet-rich fibrin)), intracanal medicament strategy (antibiotic pastes versus calcium hydroxide-based approaches), coronal barrier material (white MTA versus alternative bioceramics), and anti-discoloration procedural modifications such as access cavity management. Within each domain, randomized and comparative studies were interpreted first when available, followed by observational cohorts and case series to provide a supportive clinical context. Findings were reported using a consistent framework that considered discoloration incidence, protocol exposure, direction of effect, study design, and risk of bias. Trauma-focused cohorts were considered a distinct subgroup due to their distinct biological context and higher risk of complications affecting both esthetic and radiographic outcomes.

Results

Study Selection

**Figure 1:**
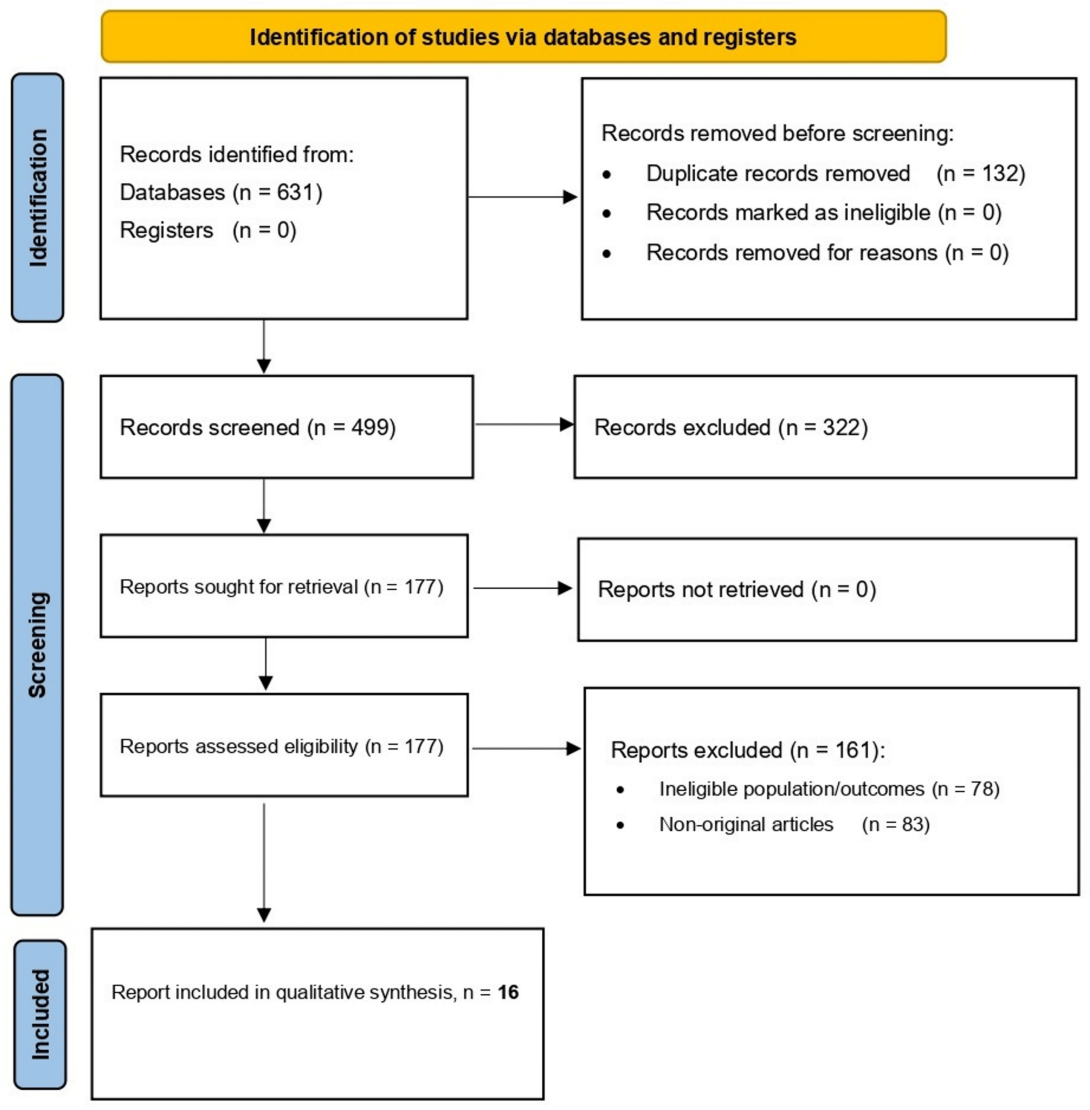
PRISMA flow summary for study identification and selection. PRISMA: Preferred Reporting Items for Systematic Reviews and Meta-Analyses.

The database search identified 631 records. After removal of duplicate records (n = 132), 499 records were screened by title and abstract, and 322 were excluded. A total of 177 full-text articles were assessed for eligibility, of which 161 were excluded; these exclusions were fully accounted for by two categories: ineligible population or outcomes (n = 78) and non-original articles (n = 83). Ultimately, 16 studies met the inclusion criteria and were included in the final qualitative synthesis (Figure 1).

Study Characteristics

The included clinical studies are listed in References [17-32]. Across the evidence base (n = 16 studies), study designs were heterogeneous and included retrospective observational cohorts, namely Choi et al. [17], Žižka et al. [18], Mittmann et al. [22], Pereira et al. [24], and Alobaid et al. [26]. These cohort studies mainly contributed evidence on real-world incidence, protocol variability, and longer-term discoloration patterns. Prospective long-term clinical studies included Jothish et al. [21] and Nazzal et al. [23]. Randomized controlled trials evaluating protocol variations and biomaterials included Tawfeek et al. [19], Rizk et al. [25], Aly et al. [27], Abd El-Hady and Badr [28], and Rizk et al. [31]. These trials primarily informed comparisons of coronal barrier materials and differences in scaffold-related protocols. Case-series level evidence included Kahler et al. [29] and Kandemir Demirci et al. [30]. One comparative clinical study was also included, namely Etman et al. [32]. Many studies involved traumatized anterior teeth, including Mittmann et al. [22], Nazzal et al. [23], Pereira et al. [24], Abd El-Hady and Badr [28], and Kahler et al. [29]. Follow-up durations ranged from 3 to 12 months in several trials, including Tawfeek et al. [19], Aly et al. [27], Abd El-Hady and Badr [28], and Rizk et al. [31], to multiyear follow-up extending beyond five years in long-term studies, including Jothish et al. [21] and Nazzal et al. [23]. This variation in follow-up duration is relevant because discoloration may be detected at different time points depending on whether it is early material-related staining or delayed perceived crown darkening. Study-level characteristics and protocol drivers relevant to discoloration are summarized in the standardized data extraction table (Table 3).

**Table 3:** Data extraction summary of included studies on tooth discoloration after regenerative endodontic therapy (RET) in immature permanent teeth (n = 16) n, number of treated teeth; N, denominator for discoloration incidence; NR, not reported; RET/REP, regenerative endodontic therapy or regenerative endodontic procedures; PRP, platelet-rich plasma; PRF, platelet-rich fibrin; A-PRF, advanced platelet-rich fibrin; WMTA, white mineral trioxide aggregate; AAEP, American Association of Endodontists.

Study (Author, Year)	Design	Teeth (n)	Follow-up	Main protocol driver relevant to discoloration	Prevention strategy reported	Discoloration incidence (n/N, %)	Key conclusion relevant to discoloration
Choi et al. (2025) [17]	Retrospective observational	42	~24 months	Protocol drivers not specified	None reported	17.9%	Discoloration occurred in a notable proportion after RET.
Žižka et al. (2021) [18]	Retrospective cohort (2 protocols)	18	Up to 24 months	Protocol differences (AAEP vs modified)	Modified protocol with access cavity sandblasting	NR (difference reported only)	Discoloration was significantly higher in AAEP protocol (p = 0.029).
Tawfeek et al. (2023) [19]	Double-blind RCT	30	12 months	Coronal barrier material (NeoMTA vs WMTA)	NeoMTA as coronal barrier	9.1% vs 27.3%	NeoMTA showed lower discoloration tendency than WMTA (between-group NS).
Narang et al. (2015) [20]	Clinical comparative study	20	6 and 18 months	Scaffold type (blood clot vs PRP vs PRF)	Not clearly specified	NR	Discoloration outcome not clearly extractable from reported data.
Jothish et al. (2024) [21]	Prospective single-arm	100	≥5 years	Long-term REP outcomes	None reported	10%	Crown discoloration occurred as a long-term complication in a minority of cases.
Mittmann et al. (2020) [22]	Retrospective cohort	16	~22 months	Trauma-focused RET	None reported	92.9%	Very high discoloration frequency after RET in traumatized incisors.
Nazzal et al. (2020) [23]	Prospective long-term	15	~61 months	Trauma-focused RET with protocol modification	Avoiding minocycline	25% patient perceived change; 33% parent perceived change	Discoloration may still occur despite efforts to reduce staining components.
Pereira et al. (2020) [24]	Retrospective cohort	16	9 to 36 months	Intracanal medicament strategy	Ca(OH)2 plus 2% chlorhexidine gel	NR	Low-staining medicament approach used, but incidence not quantified.
Rizk et al. (2020) [25]	Split-mouth double-blind RCT	NR	NR	Scaffold type (blood clot vs PRF)	PRF scaffold	NR (difference NS)	Blood clot showed more discoloration than PRF, but not statistically significant.
Alobaid et al. (2014) [26]	Retrospective cohort	31	~17 months	RET vs apexification	None reported	NR	Discoloration not reported as an extractable primary outcome.
Aly et al. (2019) [27]	RCT	26	12 months	Coronal barrier material (Biodentine vs WMTA)	Biodentine substitution	1/13 vs 7/13	WMTA was associated with higher discoloration compared with Biodentine (p = 0.01).
Abd El-Hady and Badr (2022) [28]	Randomized clinical trial	18 completed	3, 6, 12 months	Scaffold type (A-PRF vs blood clot)	A-PRF as scaffold option	NR	Discoloration was not reported as a measurable outcome in extractable form.
Kahler et al. (2014) [29]	Prospective case series	16	18 months	REP cases (protocol details limited)	None reported	10/16 unaesthetic outcomes (~62.5%)	Discoloration was a major drawback despite radiographic improvement.
Kandemir Demirci et al. (2020) [30]	Case series	3	36 months	TAP plus MTA over PRF	None reported	NR	Limited evidence with long-term healing; discoloration not quantified.
Rizk et al. (2020) [31]	Double-blinded RCT	25	12 months	Scaffold type (PRP vs PRF)	PRP vs PRF comparison	PRF higher than PRP (significant)	PRP may be preferable to PRF to reduce discoloration risk.
Etman A M etal (2020) [32]	Comparative clinical study	65	NR	Protocol subgroup variation	Not clearly specified	81.2%, 50.0%, 70.5%, 31.25%	Discoloration varied markedly across protocol subgroups.

Incidence and Pattern of Tooth Discoloration

Tooth discoloration was commonly reported across studies, but incidence varied widely due to differences in clinical protocols, materials, follow-up duration, and assessment methods. In the retrospective observational study by Choi et al. [17], discoloration occurred in 17.9% of treated teeth, providing one real-world estimate of discoloration after RET. Higher frequencies were reported in trauma-focused cohorts, where Mittmann et al. [22] observed discoloration in 92.9% of traumatized incisors after RET during approximately 22 months of follow-up. In the prospective pediatric cohort by Nazzal et al. [23], 25% of patients and 33% of parents perceived tooth color change following RET despite protocol modification, indicating that discoloration can occur even when key staining-related components are minimized. This also suggests that perceived esthetic change may differ between patients, parents, and clinical assessment methods, and should be monitored as part of RET follow-up. Long-term prospective data similarly confirmed discoloration as a clinically relevant complication, with Jothish et al. [21] reporting crown discoloration in 10% of cases over a follow-up of at least five years.

Discoloration was also identified as a notable esthetic drawback in prospective consecutive clinical cases, as shown by Kahler et al. [29], who reported unaesthetic discoloration outcomes in 10 of 16 cases (approximately 62.5%), despite documentation of periapical healing and radiographic improvements. In contrast, several studies did not quantify discoloration as an extractable incidence outcome even though they reported regenerative outcomes, including Narang et al. [20], Pereira et al. [24], Alobaid et al. [26], Abd El-Hady and Badr [28], and Kandemir Demirci et al. [30].

Protocol-Related Factors Associated With Discoloration

Coronal barrier material selection: Material selection showed a consistent directional influence on discoloration, although it should be interpreted as one component of a broader prevention protocol rather than as an isolated determinant. In a randomized clinical study, Aly et al. [27] demonstrated greater discoloration with WMTA (white mineral trioxide aggregate) than with Biodentine (7/13 vs 1/13; p = 0.01), supporting material substitution as a strategy to minimize discoloration. A similar trend was observed in Tawfeek et al. [19], where NeoMTA showed a lower discoloration frequency than WMTA (9.1% vs 27.3%), although the difference was not statistically significant. These findings support careful selection of coronal barriers, while recognizing that discoloration may also be influenced by blood contact, sealing technique, dentin exposure, chamber cleaning, and follow-up duration.

Protocol differences and access cavity management: In Žižka et al. [18], discoloration occurred significantly more often under the AAEP (American Association of Endodontists) protocol compared with a modified protocol (p = 0.029). The authors highlighted access cavity sandblasting as one component of the modified protocol, supporting the role of broader coronal dentin and access cavity management in minimizing visible staining.

Intracanal medicaments and disinfectant strategies: Etman et al. [32] demonstrated that protocol combinations can produce markedly different discoloration rates. Across four comparative subgroups, discoloration incidence was 81.2%, 50.0%, 70.5%, and 31.25%, indicating strong protocol dependency. These large differences likely reflect the combined influence of medicament choice, scaffold strategy, coronal barrier material, and sealing or chamber management rather than a single isolated protocol step. Although Pereira et al. [24] used calcium hydroxide and chlorhexidine gel, partly due to their low staining potential, the study did not report a quantified incidence of discoloration. Similarly, Narang et al. [20] and Kandemir Demirci et al. [30] used antibiotic paste-based strategies but did not report the incidence of extractable discoloration.

Scaffold type: Scaffold selection appeared to influence discoloration risk, though the direction varied across trials. In the split-mouth randomized trial by Rizk et al. [25], the blood clot group showed greater discoloration than the PRF group, but the difference was not statistically significant. In the parallel double-blind randomized trial by Rizk et al. [31], PRF resulted in significantly greater discoloration than PRP. Together, these findings suggest that scaffold-related discoloration risk may interact with co-interventions, such as intracanal medicaments, coronal barrier materials, blood-level control, and discoloration assessment methods.

Prevention strategies and evidence mapping: Prevention approaches clustered into three domains, namely substitution of staining-prone materials, access cavity and chamber management, and cautious scaffold optimization. Material substitution was the most consistently supported approach, particularly replacing WMTA with lower-staining alternatives such as Biodentine or NeoMTA, as demonstrated by Aly et al. [27] and supported by Tawfeek et al. [19]. Access cavity strategies such as sandblasting were reported to reduce discoloration in Žižka et al. [18]. Scaffold selection was a less consistent prevention domain, but remained clinically relevant because it may influence blood product exposure, chamber contamination, and interaction with coronal barrier materials. Scaffold-related prevention strategies were less consistent: Rizk et al. [25] reported lower discoloration with PRF compared with blood clot, whereas Rizk et al. [31] suggested PRP may be preferable to PRF to minimize discoloration risk. Long-term pediatric evidence also indicated that crown discoloration may persist even when minocycline is avoided, as reported by Nazzal et al. [23].

Risk of Bias Assessment

Risk of bias was assessed using design-appropriate tools: RoB 2 for randomized controlled trials and ROBINS-I for non-randomized comparative and observational studies (Table 4).

**Table 4:** Risk of bias (RoB 2) for randomized controlled trials (n = 5) RoB 2 judgments include Low risk, Some concerns, or High risk. Domains: D1, randomization process; D2, deviations from intended interventions; D3, missing outcome data; D4, measurement of the outcome; D5, selection of the reported result.

Study	Design	D1	D2	D3	D4	D5	Overall RoB
Tawfeek et al. (2023) [19]	RCT	Some concerns	Low	Some concerns	Low	Some concerns	Some concerns
Rizk et al. (2020) [25]	Split-mouth RCT	Some concerns	Low	Some concerns	Some concerns	Some concerns	Some concerns
Aly et al. (2019) [27]	RCT	Some concerns	Low	Some concerns	Low	Some concerns	Some concerns
Abd El-Hady and Badr (2022) [28]	RCT	Low	Some concerns	Some concerns	Some concerns	Some concerns	Some concerns
Rizk et al. (2020) [31]	Double-blind RCT	Some concerns	Low	Some concerns	Some concerns	Some concerns	Some concerns

Nonrandomized comparative and observational studies were evaluated using ROBINS-I across the standard domains (Table 5).

**Table 5:** Risk of bias (ROBINS-I) for non-randomized comparative and observational studies (n = 9) ROBINS-I judgments include Low, Moderate, Serious, Critical, or No information. Domains: bias due to confounding; selection of participants; classification of interventions; deviations from intended interventions; missing data; measurement of outcomes; selection of reported results.

Study	Design	Confounding	Selection	Classification	Deviations	Missing data	Outcome measurement	Selective reporting	Overall RoB
Choi et al. (2025) [17]	Retrospective	Serious	Moderate	No information	No information	Moderate	Moderate	Moderate	Serious
Žižka et al. (2021) [18]	Retrospective cohort	Serious	Moderate	No information	No information	Moderate	Moderate	Moderate	Serious
Narang et al. (2015) [20]	Clinical comparative	Moderate	Moderate	No information	No information	Moderate	Moderate	Moderate	Moderate
Jothish et al. (2024) [21]	Prospective single-arm	Serious	Moderate	Moderate	Moderate	Moderate	Moderate	Moderate	Serious
Mittmann et al. (2020) [22]	Retrospective	Serious	Moderate	No information	No information	Moderate	Moderate	Moderate	Serious
Nazzal et al. (2020) [23]	Prospective non-randomized	Serious	Moderate	No information	No information	Moderate	Moderate	Moderate	Serious
Pereira et al. (2020) [24]	Retrospective	Serious	Moderate	No information	No information	Moderate	Moderate	Moderate	Serious
Alobaid et al. (2014) [26]	Retrospective cohort	Serious	Moderate	No information	No information	Moderate	Moderate	Moderate	Serious
Etman et al. (2020) [32]	Comparative non-randomized	Serious	Moderate	No information	Moderate	Moderate	Moderate	Moderate	Serious

Case series were appraised using the Joanna Briggs Institute (JBI) Critical Appraisal Checklist for Case Series (Table 6).

**Table 6:** Quality appraisal using the JBI checklist for case series (n = 2) JBI appraisal is checklist-based and does not use RoB 2 or ROBINS-I categories. Overall appraisal reflects the balance of checklist concerns.

Study	Design	Clear inclusion criteria	Consecutive or complete inclusion	Valid outcome measurement	Adequate follow-up	Appropriate reporting and analysis	Overall appraisal
Kahler et al. (2014) [29]	Prospective case series	Some concerns	High concern	Some concerns	Some concerns	Some concerns	High concern
Kandemir Demirci et al. (2020) [30]	Case series	High concern	High concern	No information	Some concerns	Some concerns	High concern

Discussion

Overall Interpretation and Main Findings

This systematic review synthesized clinical evidence on tooth discoloration after RET and REPs in immature permanent teeth and examined how esthetic outcomes relate to clinical success and radiographic maturation. Across the included studies, RET and REPs generally achieved high clinical success and tooth survival, while the incidence of post-treatment discoloration varied markedly according to protocol factors, biomaterials, and case etiology [17-19,21-23,25,27,29,31,32]. Importantly, the available evidence suggests that clinical healing and esthetic stability do not always align. A tooth may remain asymptomatic with periapical healing while developing visible crown discoloration. This supports viewing discoloration as a parallel outcome that warrants planned esthetic monitoring and cautious prevention strategies, rather than interpreting it as treatment failure [19,22,29]. Given the heterogeneity of protocols, the absence of a meta-analysis, and concerns about risk of bias in several included studies, these findings should be interpreted as evidence-informed patterns rather than definitive causal conclusions.

Frequency of Discoloration and Sources of Heterogeneity

Across the included evidence base, reported discoloration ranged from low levels (approximately 9% to 18%) to very high rates exceeding 90%, reflecting differences in study design, protocol choices, follow-up duration, and assessment methods. In the retrospective cohort by Choi et al. [17], discoloration was observed in 17.9% of treated teeth and was reported as part of broader clinical variations after RET, providing a lower-range clinical estimate within the included evidence base. In contrast, the trauma-focused retrospective analysis by Mittmann et al. [22] documented discoloration in 92.9% of traumatized immature incisors, suggesting that severe injury contexts may amplify esthetic complications, particularly when combined with protocol factors and restorative material interactions. The prospective analysis by Kahler et al. [29] further supports the clinical relevance of this complication, reporting that crown discoloration was common and produced unaesthetic outcomes in a substantial proportion of treated teeth.

Longer follow-up studies provide additional nuance regarding perceived esthetic impact. Nazzal et al. [23] reported that 25% of patients and 33% of parents perceived color change after RET over extended follow-up, yet acceptance was often favorable. In a further long-term cohort, Jothish et al. [21] documented discoloration among complications in approximately 10% of cases in a large cohort with high overall satisfaction and survival. Together, these findings suggest that discoloration is common but context-dependent, and its reported frequency is shaped not only by biological and procedural factors but also by how outcomes are defined and perceived [21,23].

Method of Discoloration Assessment and Why Measurement Choice Matters

A key driver of heterogeneity is substantial variability in discoloration assessment. Most studies relied on visual clinician judgment or binary recording of discoloration as an adverse event [17,18,22,29,32]. In contrast, Tawfeek et al. [19] improved interpretability by combining objective spectrophotometric assessments (CIELAB and ΔE) with clinical observation and patient or parent reporting. This distinction is clinically important because measurable color shifts may remain below perceptibility thresholds, while subtle changes may be detected earlier by trained clinicians than by patients or caregivers [19,23]. Standardizing future trials around objective color measurement and consistent reporting thresholds would substantially improve comparability across protocols and materials.

Protocol Determinants of Discoloration

Coronal barrier materials as a consistent modifiable factor: Across included studies, coronal barrier material selection emerged as one of the most consistent modifiable factors associated with crown discoloration, particularly with mineral trioxide aggregate formulations. Aly et al. [27] demonstrated a clear advantage for Biodentine over white MTA, showing significantly fewer discoloration cases in the Biodentine group. Tawfeek et al. [19] similarly reported that NeoMTA exhibited a lower discoloration frequency than conventional white MTA during one-year follow-up. Although the between-group difference was not statistically significant, the direction of effect supports the clinical rationale for using newer bioceramics with improved color stability in esthetic zones [19]. These findings align with the mechanistic understanding that coronal barrier formulations can discolor teeth through interactions with dentin, blood products, and irrigants, and that radiopacifier chemistry may contribute to the observed staining patterns [19,27].

Access cavity management and cleaning: Procedural decontamination of the access cavity appears to meaningfully influence discoloration risk. Žižka et al. [18] reported significantly greater discoloration under the standard protocol than under a modified protocol incorporating additional measures, such as sandblasting. This is clinically significant because it indicates that discoloration is not purely material-dependent. It may reflect residual contamination within the pulp chamber, including blood remnants or restorative residues, that becomes visually expressed as coronal shadowing through dentin [18]. These findings support integrating structured access cavity cleaning into prevention workflows, particularly when calcium silicate-based barrier materials are used.

Scaffold selection with inconsistent direction across trials: Scaffold choice may contribute to discoloration risk, but effects were inconsistent across trials, likely reflecting interactions with co-interventions such as irrigant sequence, intracanal medicament strategy, coronal plug selection, chamber cleaning, and bleeding control. In the split-mouth RCT by Rizk et al. [25], the blood clot arm demonstrated greater crown discoloration than the PRF arm. Conversely, in the parallel, double-blind RCT by Rizk et al. [31], PRF showed significantly greater discoloration than PRP, despite both approaches achieving high short-term clinical success. Taken together, these trials suggest that platelet concentrates may influence discoloration, but the direction of benefit is protocol dependent rather than uniform [25,31].

Smaller clinical datasets provide a supportive context for platelet concentrate use but are limited for inference regarding esthetic outcomes.. Narang et al. [20] reported better regenerative outcomes with PRF than with blood clot or PRP, although discoloration was not clearly defined as a primary endpoint. Kandemir Demirci et al. [30] documented sustained healing in PRF-based RET cases with longer follow-up, but the case series design limits generalizability and precludes robust conclusions regarding discoloration frequency [30]. Overall, scaffold selection should be interpreted as one component within a comprehensive protocol rather than an isolated driver.

Intracanal medicaments, irrigants, and combined protocols: The evidence indicates that discoloration risk is multifactorial and influenced by protocol combinations. Etman et al. [32] reported wide variation in discoloration rates across subgroups of the comparative protocol, suggesting that disinfectant choice and coronal sealing strategy may substantially affect esthetic outcomes. Pereira et al. [24] used calcium hydroxide with 2% chlorhexidine gel and achieved favorable clinical and radiographic outcomes, yet discoloration persisted, underscoring that removing the risk of antibiotic-related staining does not eliminate discoloration entirely. This supports the concept that discoloration may also be driven by breakdown products of blood within dentinal tubules, cement-dentin interactions, and incomplete removal of chamber contaminants [18,19,24,32].

Clinical success versus radiographic maturation in trauma cases: Across included studies, clinical success after RET was generally high, but radiographic root maturation outcomes were more variable, particularly in trauma-dominant cohorts. Žižka et al. [18] illustrated that protocol differences can influence outcomes across domains and that clinical success does not necessarily translate into greater radiographic root area change. In the long-term cohort by Nazzal et al. [23], healing and apical closure were often observed, but root length increases were limited. Mittmann et al. [22] reported high survival in traumatized incisors despite minimal improvement in many root development parameters and a high burden of complications, including resorption and discoloration. In the retrospective dataset from Choi et al. [17], delayed root growth was frequently linked to trauma, supporting the interpretation that injury severity can compromise predictable maturogenesis [17,22,23]. Collectively, these findings reinforce that in trauma-associated necrosis, RET may deliver healing and retention more reliably than substantial root lengthening [17,22,23].

RET compared with apexification and broader clinical context: Although this review focused on discoloration after RET, comparative context remains relevant for counseling and decision-making. Alobaid et al. [26] compared revascularization with apexification and reported high survival across approaches, with adverse events occurring more frequently in the revascularization arm. This supports a balanced interpretation: RET offers biological advantages but may introduce protocol-dependent complications that require structured prevention planning, particularly when esthetics are a primary concern [26].

Clinical meaning and evidence-informed risk-minimization considerations: A consistent and clinically reassuring pattern is that discoloration does not necessarily undermine treatment success. Even in studies with high discoloration burden, overall healing and retention remained clinically meaningful [22,29]. Long-term studies further suggest that satisfaction can remain high even when color change is perceived [21,23]. Nevertheless, because immature anterior teeth often lie in the esthetic zone and involve pediatric populations, esthetic risk reduction should be considered early, particularly in trauma-related cases where risk may be compounded by structural damage and restorative complexity [22,23].

Based on the included evidence, clinicians may consider the following measures to reduce the risk of discoloration, while recognizing the limited certainty of the evidence. First, prioritize lower-staining coronal barrier materials when feasible, as NeoMTA showed a lower discoloration frequency than white MTA in an RCT [19], and Biodentine demonstrated significantly fewer discoloration events than white MTA in comparative clinical evidence [27]. Second, emphasize access cavity cleanliness, as protocol modification incorporating sandblasting was associated with reduced discoloration relative to a standard approach in Žižka et al. [18]. Third, minimize blood-cement interaction within the pulp chamber, as trial findings and mechanistic explanations indicate increased discoloration when porous calcium silicate materials contact blood products before setting [19,25,32]. Fourth, select scaffolds within a comprehensive anti-discoloration protocol rather than assuming a uniform scaffold effect, since PRF reduced discoloration relative to blood clot in one trial [25], whereas PRP reduced discoloration relative to PRF in another [31]. Finally, counsel families that esthetic change may occur even when healing is excellent, and monitor esthetic and biological endpoints separately because they may not correlate, especially in trauma-related cases [17,22,23,29].

Strengths and Limitations of the Current Evidence

Strengths of the evidence base include randomized clinical trials comparing key materials and scaffolds, which strengthen causal inference for several modifiable factors [19,25,27,28,31]. The inclusion of longer follow-up cohorts that capture patient and parent perceptions also enhances clinical relevance in real-world trauma contexts [21-23]. However, limitations remain substantial. Many studies were small, non-randomized, and relied on subjective assessment of discoloration [17,18,22,29,30,32], limiting precision and external validity. Protocol heterogeneity across medicaments, irrigants, scaffold methods, and coronal restoration strategies also complicates direct comparison. Additionally, discoloration was not consistently treated as a primary outcome, contributing to incomplete esthetic reporting in datasets focused primarily on radiographic healing [20,26,28,30].

Implications for Future Research

Future studies should standardize discoloration reporting using objective ΔE measurements, pre-specified perceptibility thresholds, and consistent follow-up intervals. Comparative designs that isolate single variables, such as barrier material, scaffold type, and chamber cleaning technique, are needed to define low-discoloration protocols without compromising regenerative biology and long-term survival [18,19,25,27,31]. Future work should also control for trauma severity, apical diameter, and patient age, which may influence both esthetic outcomes and maturogenesis [17,22,23].

## Conclusions

The included evidence supports RET and REPs as effective approaches for achieving clinical healing in immature necrotic teeth, but post-treatment discoloration remains a frequent and protocol-dependent adverse outcome. The most consistent modifiable determinants relate to coronal barrier material selection and access cavity management, whereas scaffold-related effects appear variable and protocol-dependent. Implementing evidence-aligned preventive strategies, particularly substituting lower-staining coronal plug materials and improving access cavity decontamination, offers the most defensible pathway to preserving esthetics while maintaining the biological advantages of RET.
